# Association of Female Reproductive Factors with Hypertension, Diabetes and LQTc in Chinese Women

**DOI:** 10.1038/srep42803

**Published:** 2017-02-17

**Authors:** Bayi Xu, Yequn Chen, Jianping Xiong, Nan Lu, Xuerui Tan

**Affiliations:** 1 Department of Cardiology, the First Affiliated Hospital of Shantou University Medical College, Shantou 515041, Guangdong Province, China; 2 Shantou University Medical College, Shantou, 515041, Guangdong Province, China

## Abstract

The association of female reproductive factors (FRFs) with cardiovascular risk factors among different population was variable and inconsistent. The objective of this study was to examine the association between FRFs and hypertension, type 2 diabetes mellitus (DM), and long heart-rate-corrected QT interval (LQTc) in Chinese post-menopausal women (Post-MW). A total of 8046 Post-MW from the China Chaoshan Biobank Cohort Study were included for analysis. Logistic regression and general linear regression models were used to estimate the association between FRFs and hypertension, DM, and LQTc. Compared with women with 0 or 1 live birth, increasing risk of hypertension (odds ratio [OR], 1.51; 95% confidence interval [CI], 1.16–1.96), DM (OR, 1.65; 95% CI, 1.22–2.22), and LQTc (OR, 1.45; 95% CI, 1.01–2.09) were observed in women who had five or more live births. Further analysis demonstrated that the association between parity and hypertension, DM, and LQTc was mediated by lifestyle and dyslipidemia. Women with more live births had increased body mass index and waist circumstance, and were inclined to consume more salty food, animal fat, and alcohol, but less meat, vegetable, fish, plant oil, and tea, compared with that had fewer live births (all *P* < 0.05).

Cardiovascular disease (CVD) is increasing rapidly worldwide, especially in developing countries^1^. In China, CVD is now one of the leading causes of death^2^. CVD tends to develop about 10 years later in women than in men, with a marked increase after menopause, regardless the age at which menopause occurs^3^. The most important hormonal change after menopause is the decrease of estrogen^4^, which could influence the risk of CVD through lipid and carbohydrate metabolism, coagulation parameters, blood pressure and so on^5^.

Premature menopause has been associated with elevated CVD risk^6^. Observational studies show that Post-MW using hormone replacement therapy (HRT) has a greatly reduced risk of CVD^7^. However, some large clinical randomized controlled trials found that HRT failed to decrease the risk of CVD^8^. Studies that explored the role of surrogate measures of endogenous estrogen, such as parity^9^,^10^ and age at menarche^11^,^12^, on risk of CVD also provided conflicting conclusion. Interpretations of these conflicting studies are complicated by varying control for ethnicity, socioeconomic status (SES), lifestyle, and other potentially confounding factors.

Hypertension, type 2 diabetes mellitus (hereafter referred to as diabetes mellitus, DM), and long heart-rate-corrected QT (QTc) interval (LQTc)^13^ are the established cardiovascular risk factors (CRFs) and associated with increased CVD morbidity and mortality. However, limited studies have comprehensively examined the long-term effects of reproductive history on these CRFs in Chinese women after menopause. In this paper, we took advantage of the China Chaoshan Biobank Cohort Study to examine the relationship between female reproductive factors (FRFs) and hypertension, DM, and LQTc in Post-MW. Age at menarche and menopause, parity, way of menopause, and menstrual cycle length, bleeding duration in reproductive age are the major components of FRFs in our study.

## Results

A total of 8046 Post-MW (natural menopause 7480, 93%; surgical menopause 566, 7%) were included in this study. Baseline characteristics of subjects are shown in Table 1. The overall prevalence of hypertension, DM, and LQTc among Post-MW of south China were 54.59%, 19.37%, and 9.26%, respectively, and it increased with age (Table 2).

### Association of FRFs with hypertension, DM, and LQTc

After adjusting for SES and family history of hypertension and DM, multi-party was positive associated with hypertension, DM, and LQTc. Compared with women with 0 or 1 live birth, increasing risk of hypertension (OR, 1.51; 95% CI, 1.16–1.96), DM (OR, 1.65; 95% CI, 1.22–2.22), and LQTc (OR, 1.45; 95% CI, 1.01–2.09) were observed in women who had five or more live births. Other FRFs had no association with hypertension, DM, and LQTc (see Supplementary Table S1).

In model 2, we additionally adjusted for personal hobbies and dietary habits, and found the associations between parity and hypertension, LQTc was attenuated and no longer statistically significant. Often eating meat (OR, 1.29; 95% CI, 1.07–1.57) were positive associated with hypertension, while often eating fish (OR, 0.79; 95% CI, 0.64–0.96) decreased the risk of hypertension. Tea (OR, 1.48; 95% CI, 1.07–2.05) and alcohol (OR, 0.12; 95% CI, 0.02–0.91) consumption was positive and inversely associated with increased LQTc. Multi-party was still positively associated with DM (*P* = 0.001). Often eating meat (OR, 1.34; 95% CI, 1.09–1.65) also increased the risk of DM. Smoking had no association with hypertension, DM, and LQTc (see Supplementary Table S2).

In model 3, we further adjusted body mass index (BMI), waist circumference (WC), blood lipids, and uric acid (UA) on the basis of model 2. In this model, the association between parity and DM was attenuated and no longer statistically significant. Higher BMI, WC, total cholesterol (TC), triglycerides (TG), and UA were positive associated with hypertension. Higher WC, TG, and lower high-density lipoprotein cholesterol (HDL-C) were positive associated with DM (see Supplementary Table S3).

Table 3 provides a summary of the results of logistic regression analysis (here we just listed the associations between parity and hypertension, DM, and LQTc, for other FRFs had no association with these CRFs). Decreasing trends of the association between parity and hypertension (model 1: *P* = 0.004, model 2: *P* = 0.060, and model 3: *P* = 0.196), DM (model 1: *P* < 0.0001, model 2: *P* = 0.001, and model 3: *P* = 0.057), and LQTc (model 1: *P* = 0.047, model 2: *P* = 0.067, and model 3: *P* = 0.075) were shown from model 1 to model 3.

### Association of parity with hypertension, DM, and LQTc

For other FRFs had no association with hypertension, DM, and LQTc, in this section, we just analyzed the association of parity with hypertension, DM, LQTc, and related risk factors. Women with more live births were older at enrollment and had later age at menarche and higher percentage of natural menopause (all *P* < 0.0001) than those with fewer live births (Table 4). Though women with more live births had lower percentage of family history of hypertension and DM, they still had higher prevalence of hypertension and DM (all *P* < 0.0001). The prevalence of LQTc also increased with parity (*P* < 0.0001). As for personal hobbies, multi-parity women were more likely to smoke (*P* < 0.0001) and drink (*P* = 0.021), but consumed less tea (*P* < 0.0001). Regarding dietary habits, multi-parity women were inclined to eat more salty food and animal fat, but less meat, vegetable, fish, and plant oil (all *P* < 0.0001).

General linear regression (Table 5) showed that parity were positively associated with BMI (*P* < 0.0001), WC (*P* < 0.0001), systolic blood pressure (SBP, *P* < 0.0001), diastolic blood pressure (DBP, *P* < 0.0001), fasting blood glucose (FBG, *P* < 0.0001), low-density lipoprotein cholesterol (LDL-C, *P* < 0.0001), TG (*P* < 0.05), and QTc (*P* = 0.041), and inversely associated with TC (*P* < 0.0001), HDL-C (*P* < 0.05), and UA (*P* < 0.05), after adjustment for confounders.

## Discussion

Using data from the China Chaoshan Biobank Cohort Study, we observed that the prevalence of hypertension (54.59%) and DM (19.37%) in this Post-MW was higher than that reported for women in other regions of China^14^,^15^. The most possible reason is that the participants in this study were older than those in other studies.

Menarche and menopause are two landmarks in a woman’s reproductive life. Age at menarche and menopause has been showed associated with cardiovascular risk^16^,^17^,^18^. In our study, after adjusting for SES and other FRFs, age at menarche and menopause had no association with hypertension, DM, and LQTc. Study conduct in Fujian^19^ of China also showed that no significant associations were found between age at menarche and menopause with diabetes. UK Biobank Study^20^ demonstrated that being multiple birth and premature menarche are associated with premature menopause. We found that parity was positively and negatively correlated with age at menarche and menopause respectively. The effect of age at menarche or menopause on cardiovascular risk may be affected by other factors.

Post-MW with a history of irregular menstrual cycle has been reported had more indications of CVD and were more at risk of suffering cardiovascular events and death^21^. But the association between menstrual irregularity and CRFs in our study is not significant. The possible explanation may be that our cohort recruited general Post-MW and irregular cycles are not very common and severity among them.

Compared with natural menopause women, that with surgical menopause at same ages has greater risk of CVD^6^. Women with surgical menopause in our cohort account for 7.0%, and way of menopause (natural or surgical) have no association with CRFs. Our results inconsistent with previous study probably because that surgical menopause woman only account for a small amount of the study population and had little effect on the results. Further studies of large population or case-control design are warranted.

Repeated pregnancies and deliveries may aggravate the strain to cardiovascular system in women with multi-parity^22^. Study conducted in Hubei^23^ and Guangzhou^24^ of China showed that multi-parity was associated with increasing risk of metabolic syndrome (MetS). In the present study, multi-parity was associated with increased risk of hypertension and DM. But after further adjusting for dietary habits, WC, BMI, blood lipid, and UA, the association between parity and CRFs was eliminated, suggesting these associations was mediate in part by biological changes and lifestyle factors. The average pregnancy weight gain (17.1 ± 4.9 kg) among Chinese women^25^ was much higher than the recommended^26^. Our study also showed that BMI and WC increased with parity. Body composition indices (WC or BMI) had been discovered to predict MetS in Chinese Post-MW^27^.

Whole plant foods diet is associated with decreased menopausal symptoms in Post-MW with hypertension^28^. Our study showed that women with 5 or more live births usually eat less meat, vegetable, fish, and plant oil, but more salty food and animal fat than women with fewer live births. Dietary and lifestyle influence the onset of menopause and reproductive span^29^. Healthful eating choices play a protective role in reducing the risk of developing diabetes in Post-MW^30^. Halland *et al*. also suggests that higher cardiovascular mortality associated with multi-parity is caused by accumulation of negative lifestyle factors^31^.

Ventricular arrhythmias are the main cause of sudden cardiac death^32^. An important cause of ventricular arrhythmia is prolongation of ventricular repolarization, which is reflected by a longer QT interval on electrocardiogram^33^. The prevalence of LQTc and mean QTc increased with parity in the present study. Further analysis adjusting for alcohol and tea consumption found that the association between parity and LQTc was attenuated. Alcohol consumption inversely associated with increased risk of LQTc, consistent with previous studies in the West showing that moderate alcohol use is associated with a lower risk of CVD^34^. Increased tea consumption has been found with a reduced risk of cardiovascular outcomes and total mortality^35^. Epigallocatechin-3-gallate (EGCG), major catechin of green tea, could slightly shorten the QT interval in animal model^36^. However, tea consumption was positive associated with increased risk of LQTc in our study. This is probably because first, the difference between animal and human heart; second, oolong tea is the most widely consumed tea in our study population, which different from green and black tea^37^.

The strengths of this study are the large population-based material of Post-MW. Although previous studies on FRFs mostly focused on menopause, menarche or parity, we tried to balance that inadequacy by adding menstrual cycle characteristics, way of menopause and analyzed the aforementioned synthetically. We also included careful control of abundant potential confounders in multivariate models to reduce residual confounding, which may not have been considered adequately in previous studies. Previous research on QT interval mainly focused on men, women of reproductive age or animal model, our study first analyzed LQTc in Post-MW.

There are also some limitations to our study. First, information on FRFs was self-reported and therefore misclassification is inevitable. This would tend to attenuate the observed relation between FRFs and CRFs. Second, because of the differences of regions and cultures, the results of our study cannot be automatically generalized to other population.

In summary, our study suggested that multi-parity is associated with increased risk of hypertension, DM, and LQTc. Lifestyle and dyslipidemia may play an important role in the association. Thus, prevention of CRFs in Post-MW should rely on fitness, health dietary, and treatment of hyperlipidaemia. Further studies that include more comprehensive confounders are needed to explore the unclear mechanism underlying the link between FRFs and CRFs or CVD.

## Methods

### China Chaoshan Biobank Cohort Study

The China Chaoshan Biobank Cohort Study is a large ongoing prospective, community-based study designed to investigate the prevalence of chronic non-communicable diseases and its related risk factors in south China. The study initiated in 2012 by the First Affiliated Hospital of Shantou University Medical College. Ethics approval was obtained from the Ethics Committee of the First Affiliated Hospital of Shantou University Medical College. All methods were performed in accordance with the community monitoring guidelines and regulations of the First Affiliated Hospital of Shantou University Medical College.

All participants gave informed consent prior to the study. As of December 2015, a total of 22303 participants were included in the present cohort. 8046 Post-MW from the cohort was included for the present analysis. Women who received HRT, were pregnant, had malignant tumors, or mental disorders were excluded from the study.

### Assessment of hypertension, DM, and LQTc

Hypertension was defined as systolic blood pressure (SBP) ≥140 mmHg, and/or diastolic blood pressure (DBP) ≥90 mmHg, and/or use of antihypertensive medications^38^. Type 2 diabetes mellitus was defined as fasting blood glucose (FBG) ≥7 mmol/L (126 mg/dL) and/or being on treatment for diabetes^39^.

Resting heart rates and QT intervals were obtained from the electrocardiograms (ECGs). QT corrected for the length of the previous cycle (QTc) was obtained using Bazett’s formulae^40^. QTc ≥460 ms was defined as abnormally prolonged.

### Assessment of female reproductive factors

Postmenopausal was defined as lack of menstrual bleeding for at least 12 months or a history of hysterectomy and oophorectomy^41^. Age at menarche and menopause was retrospectively assigned as the self-reported age at the first and last menstrual period. Menstrual cycle length in reproductive age was referred as the number of days from the start of one bleeding period through the day before the start of the next menses. Bleeding duration in reproductive age was referred as the number of days from the start of one menstrual bleeding period through the last day of bleeding. The number of live births was referred to as parity in this paper. The ways of menopause include natural and surgical ways.

### Assessment of covariates

Standing height, weight, WC, TC, LDL-C, HDL-C, TG, and UA were obtained from the medical examination. BMI was defined as weight (kg)/height^2^ (m^2^). BMI and WC were divided into three groups according to the guidelines for prevention and control of overweight and obesity in Chinese adults^42^.

Age at enrollment, marital status, occupational type, highest educational attainment, personal and family medical history (including hypertension and diabetes), FRFs, personal hobbies (smoking, alcohol and tea consumption), and dietary habits were collected via questionnaire-based interview. Family medical history included diseases in first-degree relatives including parents, offspring, and siblings. Dietary habits include the eating frequency (sometimes and often) of meat, viscus, vegetable, fish, salty food, plant oil, and animal fat. Current smoking was defined as smoking at least once a day over a period of more than half a year. Current alcohol and tea consumption was defined as drinking alcohol or tea once a week for a period of more than half a year.

### Statistical analysis

All statistical analyses were performed in SPSS 19.0 (IBM Corp© 2010) and the probability level accepted for significance was *P* < 0.05.

Logistic regressions were used to examine the associations between FRFs and hypertension, DM, and LQTc. Results are presented as odds ratios (ORs) with 95% confidence intervals (CIs).

The differences among subjects in different groups were detected using the Kruskal-Wallis test for continuous variables and the *χ*^2^ test for categorical variables. A post hoc test using Bonferroni correction was applied, given that the overall test was significant (*P* < 0.05). Continuous variables were summarized by medians with interquartile ranges (IQRs) (the range between the 25th and 75th percentile). Categorical variables were expressed by percentages.

General linear regression models were created to examine the association of continuous parity (exposure) with each measure of cardiovascular risk factors (outcome variables). Results are presented as least square means (LSMs) with 95% CIs and *P* values comparing differences across parity were obtained from the models.

## Additional Information

**How to cite this article**: Xu, B. *et al*. Association of Female Reproductive Factors with Hypertension, Diabetes and LQTc in Chinese Women. *Sci. Rep.* **7**, 42803; doi: 10.1038/srep42803 (2017).

**Publisher's note:** Springer Nature remains neutral with regard to jurisdictional claims in published maps and institutional affiliations.

## Supplementary Material

Supplementary Information

## Figures and Tables

**Table 1:** Baseline characteristics of study participants.

Characteristics	No. of survey (%)	Characteristics	No. of survey (%)	Characteristics	No. of survey (%)
Parity, live births		61–70	3084 (38.3)	Meat	
0–1 (Ref.)	876 (10.9)	≥71	1321 (16.4)	Sometimes	5174 (64.3)
2	1595 (19.8)	Occupational type		Often	2846 (35.4)
3	2227 (27.7)	Manual	1075 (13.4)	Missing	26 (0.3)
4	1550 (19.3)	Mental	387 (4.8)	Viscus	
≥5	1798 (22.3)	Housework/retirement	6505 (80.8)	Sometimes	7343 (91.3)
Age at menarche, y		Other	66 (0.8)	Often	693 (8.6)
≤14	1097 (13.6)	Missing	13 (0.2)	Missing	10 (0.1)
15	1302 (16.2)	Marital status		Vegetable	
16 (Ref.)	1404 (17.4)	Married	6883 (85.5)	Sometimes	2897 (36.0)
17	1402 (17.4)	Widowed/divorced	1126 (14.0)	Often	5129 (63.8)
18	1275 (15.8)	Missing	37 (0.5)	Missing	20 (0.2)
≥19	1511 (18.8)	Highest educational attainment		Fish	
Missing	55 (0.7)	No formal	3410 (42.4)	Sometimes	4897 (60.9)
Age at menopause, y		Primary school	2883 (35.8)	Often	3094 (38.5)
≤47	1458 (18.1)	Junior high school	703 (8.7)	Missing	55 (0.7)
48–49	1541 (19.2)	Senior middle school	730 (9.1)	Salty food	
50 (Ref.)	1351 (16.8)	College or above	308 (3.8)	Sometimes	5220 (64.9)
51–52	1581 (19.6)	Missing	12 (0.1)	Often	2786 (34.6)
53–54	1109 (13.8)	Family history of hypertension		Missing	40 (0.5)
≥55	957 (11.9)	No	6634 (82.5)	Plant oil	
Missing	48 (0.6)	Yes	1412 (17.5)	Sometimes	4081 (50.7)
Menstrual cycle length, d		Family history of diabetes		Often	3932 (48.9)
≤27	300 (3.7)	No	7490 (93.1)	Missing	33 (0.4)
28–30 (Ref.)	2189 (27.2)	Yes	556 (6.9)	Animal fat	
≥31	5518 (68.6)	Smoking		Sometimes	6159 (76.5)
Missing	39 (0.5)	Non	7429 (92.3)	Often	1868 (23.2)
Bleeding duration, d		Current	587 (7.3)	Missing	19 (0.3)
≤3	1528 (19.0)	Missing	30 (0.4)	BMI, kg/m^2^	
4–6 (Ref.)	4932 (61.3)	Alcohol consumption		<24	3718 (46.2)
≥7	1556 (19.3)	Non	7825 (97.3)	24–27	3117 (38.7)
Missing	30 (0.4)	Current	183 (2.3)	≥28	1165 (14.5)
Way of menopause		Missing	38 (0.5)	Missing	46 (0.6)
Natural	7480 (93.0)	Tea consumption		Waist circumference, cm	
Surgical	566 (7.0)	Non	1855 (23.1)	<80	2616 (32.5)
Age at enrollment, y		Current	6163 (76.6)	80–89	3222 (40)
≤50	454 (5.6)	Missing	28 (0.3)	≥90	2155 (26.8)
51–60	3184 (39.6)			Missing	53 (0.7)

Abbreviations: y, years; d, days; BMI, body mass index.

**Table 2:** The prevalence of hypertension, DM, and LQTc in post-menopausal women of China (%).

Age at enrollment	Hypertension	DM	LQTc
≤50	33.41	10.35	4.76
51–60	44.73*	16.05*	8.19*
61–70	60.31*^†^	22.24*^†^	9.42 *
≥71	72.30*^†‡^	23.77*^†^	13.02*^†‡^
Total	54.59	19.37	9.26
*P* for trend	<0.0001	<0.0001	<0.0001

*Compared with age ≤ 50, P < 0.05. ^†^Compared with age 51–60, P < 0.05. ^‡^Compared with age 61–70, P < 0.05. Trend test was examined by χ^2^ test (Linear-by-Linear Association). Abbreviations: DM, type 2 diabetes mellitus; LQTc, long heart-rate-corrected QT interval. Difference between age groups was examined by χ^2^ test.

**Table 3:** Odds ratios (95% confidence intervals) of hypertension, DM, and LQTc according to parity categories in different logistic regression models.

Models	0–1 (Ref.)	2	3	4	≥5	*P* value
		OR (95% CI)	OR (95% CI)	OR (95% CI)	OR (95% CI)	
Hypertension						
Model 1	1.00	1.20 (0.98, 1.48)	1.40 (1.13, 1.75)	1.55 (1.22, 1.95)	1.51 (1.16, 1.96)	0.004
Model 2	1.00	1.06 (0.81, 1.38)	1.20 (0.91, 1.58)	1.39 (1.04, 1.87)	1.46 (1.06, 2.02)	0.060
Model 3	1.00	0.94 (0.69, 1.27)	1.01 (0.73, 1.39)	1.21 (0.86, 1.70)	1.29 (0.89, 1.89)	0.196
DM						
Model 1	1.00	1.28 (1.00, 1.64)	1.70 (1.32, 2.20)	1.49 (1.13, 1.95)	1.65 (1.22, 2.22)	<0.0001
Model 2	1.00	1.26 (0.92, 1.74)	1.68 (1.21, 2.34)	1.47 (1.04, 2.07)	1.58 (1.09, 2.29)	0.001
Model 3	1.00	1.17 (0.82, 1.67)	1.55 (1.08, 2.24)	1.35 (0.92, 1.98)	1.33 (0.88, 2.03)	0.057
LQTc						
Model 1	1.00	1.15 (0.85, 1.55)	1.19 (0.87, 1.64)	1.25 (0.87, 1.81)	1.45 (1.01, 2.09)	0.047
Model 2	1.00	1.28 (0.86, 1.89)	1.39 (0.92, 2.09)	1.57 (0.99, 2.49)	1.53 (0.96, 2.44)	0.067
Model 3	1.00	1.22 (0.75, 1.97)	1.23 (0.74, 2.04)	1.74 (1.01, 2.98)	1.54 (0.89, 2.66)	0.075

Model 1: age at menarche, age at menopause, cycle length, bleeding duration, way of menopause, age at enrollment, marital status, occupational type, highest educational attainment, family history of hypertension, family history of diabetes; Model 2: model 1 + smoking, alcohol consumption, tea consumption, meat, viscus, vegetable, fish, salty food, plant oil, animal fat; Model 3: model 2 + BMI, WC, TC, TG, UA, LDL-C, HDL-C. Abbreviations: DM, type 2 diabetes mellitus; LQTc, long heart-rate-corrected QT interval.

**Table 4:** The differences among subjects by parity (live births).

Variables		0–1	2	3	4	≥5	*P* value
Age at enrollment, y^#^		58 (54, 62)	60 (55, 64)*	62 (58, 66)*^†^	63 (58, 69)*^†‡^	68 (57, 77)*^†‡§^	<0.0001
FRFs	Age at menarche, y^#^	15 (14, 16)	16 (15, 18)*	17 (15, 18)*^†^	17 (16, 18)*^†‡^	17 (16, 18)*^†‡^	<0.0001
	Age at menopause, y^#^	50 (48, 53)	50 (48, 53)	50 (48, 53)	50 (48, 52)*^†‡^	50 (47, 52)*^†‡^	0.010
	MCL, d^#^	30 (30, 30)	30 (28, 30)*	30 (28, 30)*	30 (28, 30)*	30 (28, 30)*	<0.0001
	Bleeding duration, d^#^	5 (4, 7)	5 (4, 6)*	5 (4, 6)*^†^	5 (4, 6)*^†^	5 (4, 6)*^†^	<0.0001
	Natural menopause, %**	87.56	90.72*	93.53*^†^	94.52*^†^	95.74*^†‡^	<0.0001
Hypertension**	Prevalence, %	39.49	49.02*	55.57*^†^	59.28*^†^‡	62.25*^†‡^	<0.0001
	Family history, %	37.67	21.38*	14.68*^†^	14.58*^†^	9.54*^†‡§^	<0.0001
DM**	Prevalence, %	9.59	14.67*	22.00*^†^	19.74*^†^	25.59*^†‡§^	<0.0001
	Family history, %	18.84	8.28*	4.98*^†^	4.71*^†^	3.82*^†^	<0.0001
LQTc, %**		3.46	7.04*	9.65*^†^	9.68*^†^	12.76*^†‡§^	<0.0001
Personal Hobbies**	Smoking, %	1.73	4.08*	7.74*^†^	9.58*^†^	11.01*^†‡^	<0.0001
	Alcohol consumption, %	1.27	1.76	2.34	2.20	3.26*^†^	0.021
	Tea consumption, %	84.66	79.30*	76.46*^†^	75.28*^†^	72.58*^†‡^	<0.0001
Dietary habits (often eating)**	Meat, %	36.45	37.37	37.95	36.15	27.18^†‡§^	<0.0001
	Viscus, %	7.94	7.51	8.11	9.35	10.77^†‡^	0.065
	Vegetable, %	62.36	66.37	66.56*	64.66	53.49*^†‡§^	<0.0001
	Fish, %	37.11	38.58	41.55*	41.26	30.52*^†‡§^	<0.0001
	Salty food, %	14.25	30.40*	37.95*^†^	38.99*^†^	43.41*^†‡§^	<0.0001
	Plant oil, %	84.01	61.19*	43.98*^†^	39.51*^†‡^	32.60*^†‡§^	<0.0001
	Animal fat, %	8.05	19.70*	26.99*^†^	25.88*^†^	26.33*^†^	<0.0001

^#^Continuous data are presented as median and interquartile range (IQR) and were examined by the Kruskal-Wallis test. **Categorical data are presented as percentage (%) and were examined by χ^2^ test. *Compared with 0–1 live birth, *P* < 0.05. ^†^Compared with 2 live births, *P* < 0.05; ^‡^compared with 3 live births, *P* < 0.05; ^§^compared with 4 live births, *P* < 0.05; Abbreviations: y, years; d, days; MCL, menstrual cycle length; DM, type 2 diabetes mellitus; LQTc, long heart-rate-corrected QT interval.

**Table 5:** Least squares means (95% confidence intervals) of cardiovascular risk factors according to parity categories in different general linear regression models.

Variables	Model	0–1	2	3	4	≥5	*P* value
BMI, kg/m^2^	Model 1	23.6 (23.3, 23.8)	24.2 (24.0, 24.3)	24.7 (24.5, 24.8)	24.7 (24.5, 24.9)	25.0 (24.7, 25.2)	<0.0001
	Model 2	23.4 (23.1, 23.6)	24.2 (24.0, 24.4)	24.8 (24.6, 24.9)	24.9 (24.7, 25.1)	25.1 (24.8, 25.4)	<0.0001
WC, cm	Model 1	81 (80, 81)	82 (82, 83)	85 (84, 85)	84 (84, 85)	85 (84, 86)	<0.0001
	Model 2	80 (79, 81)	82 (81, 82)	85 (84, 85)	85 (84, 85)	85 (84, 86)	<0.0001
SBP, mm Hg	Model 1	131 (129, 132)	135 (134, 136)	137 (136, 138)	137 (136, 138)	138 (136, 139)	<0.0001
	Model 2	130 (128, 132)	134 (133, 135)	136 (135, 137)	136 (135, 137)	138 (136, 139)	<0.0001
DBP, mm Hg	Model 1	80 (79, 81)	83 (83, 84)	85 (84, 85)	85 (84, 85)	84 (84, 85)	<0.0001
	Model 2	80 (79, 81)	83 (83, 84)	85 (84, 85)	84 (84, 85)	85 (84, 85)	<0.0001
FBG, mmol/L	Model 1	5.51 (5.36, 5.67)	5.94 (5.83, 6.05)	6.20 (6.11, 6.29)	6.02 (5.91, 6.13)	6.32 (6.18, 6.47)	<0.0001
	Model 2	5.51 (5.34, 5.68)	5.91 (5.79, 6.02)	6.19 (6.09, 6.29)	5.99 (5.87, 6.11)	6.36 (6.18, 6.53)	<0.0001
TC, mmol/L	Model 1	6.18 (6.09, 6.26)	5.99 (5.92, 6.05)	5.95 (5.90, 6.00)	5.92 (5.86, 5.98)	5.84 (5.76, 5.93)	<0.0001
	Model 2	6.16 (6.07, 6.26)	5.97 (5.90, 6.03)	5.95 (5.89, 6.00)	5.91 (5.85, 5.98)	5.84 (5.75, 5.94)	<0.0001
HDL-C, mmol/L	Model 1	1.41 (1.39, 1.43)	1.43 (1.41, 1.44)	1.40 (1.39, 1.42)	1.41 (1.39, 1.43)	1.38 (1.36, 1.40)	0.018
	Model 2	1.41 (1.39, 1.44)	1.43 (1.41, 1.44)	1.40 (1.39, 1.42)	1.41 (1.39, 1.42)	1.38 (1.35, 1.40)	0.020
LDL-C, mmol/L	Model 1	2.79 (2.72, 2.86)	3.37 (3.32, 3.42)	3.66 (3.61, 3.71)	3.65 (3.59, 3.70)	3.72 (3.65, 3.79)	<0.0001
	Model 2	2.77 (2.70, 2.85)	3.35 (3.29, 3.40)	3.65 (3.60, 3.70)	3.64 (3.58, 3.70)	3.69 (3.61, 3.77)	<0.0001
TG, mmol/L	Model 1	1.57 (1.46, 1.69)	1.83 (1.75, 1.91)	1.80 (1.73, 1.86)	1.76 (1.67, 1.84)	1.88 (1.78, 1.99)	0.002
	Model 2	1.61 (1.49, 1.73)	1.81 (1.73, 1.90)	1.78 (1.71, 1.86)	1.74 (1.65, 1.83)	1.90 (1.78, 2.03)	0.020
UA, μmol/L	Model 1	336 (329, 343)	322 (317, 327)	320 (316, 324)	311 (306, 316)	313 (306, 319)	<0.0001
	Model 2	331 (323, 338)	320 (315, 326)	319 (315, 324)	314 (308, 319)	313 (305, 321)	0.015
QTc, ms	Model 1	416 (413, 419)	416. (414, 418)	418 (416, 419)	418 (416, 420)	421 (419, 424)	0.041
	Model 2	416 (413, 419)	416 (414, 418)	417 (415, 418)	419 (417, 421)	421 (418, 424)	0.122

Model 1: age at enrollment; Model 2: model 1 + age at menarche, age at menopause, menstrual cycle length and bleeding duration. *P* values were reported for parity as a linear variable (ordinal scale: 1 as group of 0−1; 2 as group of 2; 3 as group of 3; 4 as group of 4; and 5 as group of ≥5). Abbreviations: BMI, body mass index; WC, Waist circumference; SBP, systolic blood pressure; DBP, diastolic blood pressure; FBG, fasting blood glucose; TC, total cholesterol; HDL-C, high-density lipoprotein cholesterol; LDL-C, low-density lipoprotein cholesterol; TG, triglycerides; UA, uric acid; QTc, heart-rate-corrected QT interval.
