# Supplementary material for: Association of Female Reproductive Factors with Hypertension, Diabetes and LQTc in Chinese Women
Source: Sci Rep. 2017 Feb 17;7:42803. doi: 10.1038/srep42803 (PMC5314360; doi:10.1038/srep42803)
Supplement: Supplementary Information [file srep42803-s1.doc]

**Association of Female Reproductive Factors with Hypertension,**

**Diabetes, and LQTc in Chinese Women**

Bayi Xu^1,2^, Yequn Chen^1^, Jianping Xiong^1^, Nan Lu^1^, Xuerui Tan^1^

^1^Department of Cardiology, the First Affiliated Hospital of Shantou University Medical College, Shantou 515041, Guangdong Province, China. ^2^Shantou University Medical College, Shantou, 515041, Guangdong Province, China. Correspondence and requests for materials should be addressed to X.T. (email: [tanxuerui@vip.sina.com](mailto:tanxuerui@vip.sina.com))

| Variable | | Hypertension | | DM | | LQTc | |
| --- | --- | --- | --- | --- | --- | --- | --- |
|  |  | *p* | OR (95% CI) | *p* | OR (95% CI) | *p* | OR (95% CI) |
| Parity, live births | 0-1 | 0.004 |  | < 0.0001 |  | 0.047 |  |
|  | 2 | 0.084 | 1.20 (0.98, 1.48) | 0.054 | 1.28 (1.00, 1.64) | 0.354 | 1.15 (0.85, 1.55) |
|  | 3 | 0.002 | 1.40 (1.13, 1.75) | < 0.0001 | 1.70 (1.32, 2.20) | 0.277 | 1.19 (0.87, 1.64) |
|  | 4 | < 0.0001 | 1.55 (1.22, 1.95) | 0.004 | 1.49 (1.13, 1.95) | 0.230 | 1.25 (0.87, 1.81) |
|  | ≥ 5 | 0.002 | 1.51 (1.16, 1.96) | 0.001 | 1.65 (1.22, 2.22) | 0.046 | 1.45 (1.01, 2.09) |
| Age at menarche, y | 16 | 0.214 |  | 0.009 |  | 0.686 |  |
|  | ≤ 14 | 0.623 | 0.95 (0.78, 1.16) | 0.264 | 1.13 (0.91, 1.39) | 0.858 | 0.96 (0.64, 1.44) |
|  | 15 | 0.629 | 1.05 (0.87, 1.26) | 0.241 | 1.13 (0.92, 1.37) | 0.503 | 0.88 (0.61, 1.28) |
|  | 17 | 0.269 | 0.90 (0.76, 1.08) | 0.074 | 0.84 (0.69, 1.02) | 0.395 | 1.15 (0.83, 1.59) |
|  | 18 | 0.921 | 0.99 (0.82, 1.19) | 0.669 | 1.04 (0.86, 1.27) | 0.664 | 1.08 (0.77, 1.50) |
|  | ≥ 19 | 0.054 | 0.84 (0.70, 1.00) | 0.098 | 0.85 (0.70, 1.03) | 0.408 | 1.14 (0.83, 1.57) |
| Age at menopause, y | 50 | 0.216 |  | 0.159 |  | 0.380 |  |
|  | ≤ 47 | 0.392 | 0.92 (0.77, 1.11) | 0.573 | 0.94 (0.78, 1.15) | 0.356 | 0.85 (0.61, 1.20) |
|  | 48-49 | 0.514 | 1.06 (0.89, 1.27) | 0.516 | 1.07 (0.88, 1.29) | 0.479 | 0.89 (0.64, 1.23) |
|  | 51-52 | 0.886 | 1.01 (0.85, 1.21) | 0.121 | 0.86 (0.71, 1.04) | 0.297 | 1.18 (0.86, 1.62) |
|  | 53-54 | 0.264 | 1.11 (0.92, 1.35) | 0.372 | 1.10 (0.89, 1.35) | 0.887 | 1.03 (0.72, 1.45) |
|  | ≥ 55 | 0.124 | 1.17 (0.96, 1.43) | 0.897 | 1.01 (0.82, 1.26) | 0.739 | 0.94 (0.64, 1.36) |
| Cycle length, d | 28-30 | 0.206 |  | 0.027 |  | 0.963 |  |
|  | ≤ 27 | 0.075 | 0.80 (0.63, 1.02) | 0.048 | 0.75 (0.56, 1.00) | 0.805 | 0.94 (0.58, 1.53) |
|  | ≥ 31 | 0.930 | 0.99 (0.79, 1.23) | 0.089 | 1.22 (0.97, 1.54) | 0.916 | 1.02 (0.69, 1.51) |
| Bleeding duration, d | 4-6 | 0.316 |  | 0.287 |  | 0.070 |  |
|  | ≤ 3 | 0.388 | 1.06 (0.92, 1.23) | 0.937 | 1.01 (0.87, 1.17) | 0.149 | 1.19 (0.94, 1.52) |
|  | ≥ 7 | 0.309 | 0.93 (0.81, 1.07) | 0.129 | 0.89 (0.76, 1.03) | 0.159 | 0.82 (0.62, 1.08) |
| Way of menopause | | 0.897 | 1.01 (0.81, 1.27) | 0.174 | 1.18 (0.93, 1.51) | 0.763 | 1.07 (0.69, 1.66) |
| Age at enrollment | | < 0.0001 | 1.74 (1.61, 1.88) | < 0.0001 | 1.25 (1.15, 1.36) | 0.053 | 1.15 (1.00, 1.32) |
| Marital status | | 0.035 | 1.22 (1.01, 1.46) | 0.181 | 0.88 (0.73, 1.06) | 0.829 | 1.03 (0.77, 1.39) |
| Occupational type | | 0.263 | 0.96 (0.89, 1.03) | 0.051 | 1.09 (1.00, 1.18) | 0.038 | 0.88 (0.77, 0.99) |
| Highest educational attainment | | < 0.0001 | 0.84 (0.79, 0.90) | < 0.0001 | 0.87 (0.81, 0.93) | 0.001 | 0.81 (0.71, 0.92) |
| Family history of hypertension | | < 0.0001 | 2.41 (2.08, 2.79) | 0.653 | 0.96 (0.82, 1.13) | 0.453 | 1.11 (0.84, 1.46) |
| Family history of diabetes | | 0.297 | 0.90 (0.73, 1.10) | < 0.0001 | 2.92 (2.37, 3.60) | 0.702 | 0.92 (0.58, 1.44) |

**Supplementary Table S1. Logistic regression analysis of associations between FRFs and hypertension, DM, and LQTc (model 1).** Abbreviations: y, years; d, days; FRFs, female reproductive factors; DM, type 2 diabetes mellitus; LQTc, long heart-rate-corrected QT interval.

| Variable | | Hypertension | | DM | | LQTc | |
| --- | --- | --- | --- | --- | --- | --- | --- |
|  |  | *p* | OR (95% CI) | *p* | OR (95% CI) | *p* | OR (95% CI) |
| Parity, live births | 0-1 | 0.060 |  | 0.001 |  | 0.067 |  |
|  | 2 | 0.676 | 1.06 (0.81, 1.38) | 0.151 | 1.26 (0.92, 1.74) | 0.222 | 1.28 (0.86, 1.89) |
|  | 3 | 0.208 | 1.20 (0.91, 1.58) | 0.002 | 1.68 (1.21, 2.34) | 0.120 | 1.39 (0.92, 2.09) |
|  | 4 | 0.026 | 1.39 (1.04, 1.87) | 0.027 | 1.47 (1.04, 2.07) | 0.057 | 1.57 (0.99, 2.49) |
|  | ≥ 5 | 0.021 | 1.46 (1.06, 2.02) | 0.015 | 1.58 (1.09, 2.29) | 0.071 | 1.53 (0.96, 2.44) |
| Age at menarche, y | 16 | 0.279 |  | 0.064 |  | 0.585 |  |
|  | ≤ 14 | 0.968 | 1.00 (0.79, 1.26) | 0.229 | 1.17 (0.91, 1.50) | 0.598 | 1.13 (0.71, 1.82) |
|  | 15 | 0.243 | 1.14 (0.91, 1.42) | 0.369 | 1.11 (0.88, 1.41) | 0.518 | 0.86 (0.54, 1.36) |
|  | 17 | 0.671 | 0.95 (0.77, 1.18) | 0.082 | 0.81 (0.65, 1.03) | 0.419 | 1.18 (0.79, 1.74) |
|  | 18 | 0.796 | 0.97 (0.78, 1.21) | 0.904 | 0.99 (0.78, 1.25) | 0.818 | 0.95 (0.62, 1.45) |
|  | ≥ 19 | 0.149 | 0.85 (0.68, 1.06) | 0.355 | 0.90 (0.71, 1.13) | 0.330 | 1.21 (0.82, 1.80) |
| Age at menopause, y | 50 | 0.225 |  | 0.187 |  | 0.633 |  |
|  | ≤ 47 | 0.186 | 0.86 (0.69, 1.07) | 0.487 | 0.92 (0.72, 1.17) | 0.158 | 0.74 (0.48, 1.13) |
|  | 48-49 | 0.572 | 1.06 (0.86, 1.32) | 0.494 | 1.08 (0.86, 1.36) | 0.684 | 0.92 (0.62, 1.37) |
|  | 51-52 | 0.612 | 0.95 (0.77, 1.17) | 0.163 | 0.85 (0.67, 1.07) | 0.785 | 0.95 (0.64, 1.41) |
|  | 53-54 | 0.527 | 1.08 (0.86, 1.36) | 0.677 | 1.05 (0.82, 1.35) | 0.682 | 1.09 (0.71, 1.67) |
|  | ≥ 55 | 0.362 | 1.12 (0.88, 1.44) | 0.421 | 1.11 (0.86, 1.44) | 0.828 | 0.95 (0.60, 1.51) |
| Cycle length, d | 28-30 | 0.063 |  | 0.057 |  | 0.905 |  |
|  | ≤ 27 | 0.004 | 0.64 (0.47, 0.87) | 0.147 | 0.77 (0.54, 1.10) | 0.884 | 1.05 (0.58, 1.89) |
|  | ≥ 31 | 0.777 | 0.96 (0.74, 1.25) | 0.072 | 1.28 (0.98, 1.67) | 0.665 | 1.11 (0.70, 1.75) |
| Bleeding duration, d | 4-6 | 0.109 |  | 0.228 |  | 0.071 |  |
|  | ≤ 3 | 0.058 | 1.18 (0.99, 1.40) | 0.997 | 1.00 (0.84, 1.20) | 0.090 | 1.29 (0.96, 1.73) |
|  | ≥ 7 | 0.637 | 0.96 (0.81, 1.14) | 0.094 | 0.85 (0.71, 1.03) | 0.264 | 0.82 (0.58, 1.16) |
| Way of menopause | | 0.514 | 1.09 (0.84, 1.43) | 0.060 | 1.31 (0.99, 1.75) | 0.224 | 1.36 (0.83, 2.25) |
| Age at enrollment | | < 0.0001 | 1.77 (1.60, 1.95) | < 0.0001 | 1.24 (1.12, 1.38) | 0.664 | 0.96 (0.80, 1.15) |
| Marital status | | 0.084 | 1.22 (0.97, 1.52) | 0.894 | 1.02 (0.81, 1.27) | 0.371 | 1.19 (0.82, 1.72) |
| Occupational type | | 0.386 | 0.96 (0.86, 1.06) | 0.026 | 1.14 (1.02, 1.27) | 0.332 | 0.92 (0.77, 1.09) |
| Highest educational attainment | | < 0.0001 | 0.82 (0.76, 0.89) | < 0.0001 | 0.85 (0.78, 0.92) | 0.018 | 0.81 (0.68, 0.96) |
| Family history of hypertension | | < 0.0001 | 2.33 (1.95, 2.79) | 0.736 | 0.97 (0.80, 1.17) | 0.497 | 1.12 (0.80, 1.57) |
| Family history of diabetes | | 0.103 | 0.80 (0.62, 1.04) | < 0.0001 | 3.25 (2.50, 4.24) | 0.975 | 1.01 (0.58, 1.76) |
| Smoking | | 0.076 | 0.79 (0.61, 1.03) | 0.498 | 0.91 (0.69, 1.20) | 0.793 | 1.06 (0.68, 1.64) |
| Alcohol consumption | | 0.530 | 1.16 (0.73, 1.86) | 0.823 | 1.06 (0.65, 1.71) | 0.040 | 0.12 (0.02, 0.91) |
| Tea consumption | | 0.929 | 1.01 (0.86, 1.19) | 0.384 | 1.08 (0.91, 1.29) | 0.019 | 1.48 (1.07, 2.05) |
| Meat | | 0.009 | 1.29 (1.07, 1.57) | 0.006 | 1.34 (1.09, 1.65) | 0.275 | 0.82 (0.57, 1.17) |
| Viscus | | 0.514 | 1.09 (0.85, 1.40) | 0.304 | 0.86 (0.66, 1.14) | 0.103 | 0.64 (0.38, 1.09) |
| Vegetable | | 0.301 | 1.09 (0.93, 1.27) | 0.703 | 0.97 (0.82, 1.15) | 0.231 | 0.83 (0.62, 1.12) |
| Fish | | 0.018 | 0.79 (0.64, 0.96) | 0.131 | 0.85 (0.68, 1.05) | 0.558 | 1.12 (0.77, 1.62) |
| Salty Food | | 0.498 | 1.05 (0.91, 1.22) | 0.810 | 0.98 (0.84, 1.14) | 0.290 | 1.15 (0.89, 1.49) |
| Plant oil | | 0.507 | 1.05 (0.91, 1.21) | 0.439 | 1.06 (0.91, 1.23) | 0.171 | 0.83 (0.64, 1.08) |
| Animal fat | | 0.131 | 0.88 (0.76, 1.04) | 0.146 | 0.88 (0.74, 1.04) | 0.954 | 1.01 (0.76, 1.33) |

**Supplementary Table S2. Logistic regression analysis of associations between FRFs and hypertension, DM, and LQTc (model 2).** Abbreviations: y, years; d, days; FRFs, female reproductive factors; DM, type 2 diabetes mellitus; LQTc, long heart-rate-corrected QT interval.

| Variable | | Hypertension | | DM | | LQTc | |
| --- | --- | --- | --- | --- | --- | --- | --- |
|  |  | *p* | OR (95% CI) | *p* | OR (95% CI) | *p* | OR (95% CI) |
| Parity, live births | 0-1 | 0.196 |  | 0.057 |  | 0.075 |  |
|  | 2 | 0.682 | 0.94 (0.69, 1.27) | 0.383 | 1.17 (0.82, 1.67) | 0.425 | 1.22 (0.75, 1.97) |
|  | 3 | 0.970 | 1.01 (0.73, 1.39) | 0.019 | 1.55 (1.08, 2.24) | 0.426 | 1.23 (0.74, 2.04) |
|  | 4 | 0.282 | 1.21 (0.86, 1.70) | 0.126 | 1.35 (0.92, 1.98) | 0.045 | 1.74 (1.01, 2.98) |
|  | ≥ 5 | 0.181 | 1.29 (0.89, 1.89) | 0.176 | 1.33 (0.88, 2.03) | 0.127 | 1.54 (0.89, 2.66) |
| Age at menarche, y | 16 | 0.814 |  | 0.180 |  | 0.683 |  |
|  | ≤ 14 | 0.582 | 0.93 (0.71, 1.21) | 0.591 | 1.08 (0.81, 1.44) | 0.767 | 1.09 (0.63, 1.88) |
|  | 15 | 0.328 | 1.14 (0.88, 1.46) | 0.312 | 1.15 (0.88, 1.51) | 0.913 | 0.97 (0.57, 1.66) |
|  | 17 | 0.816 | 1.03 (0.81, 1.32) | 0.103 | 0.80 (0.61, 1.05) | 0.384 | 1.23 (0.77, 1.96) |
|  | 18 | 0.735 | 1.05 (0.80, 1.36) | 0.716 | 1.05 (0.80, 1.39) | 0.542 | 0.85 (0.50, 1.44) |
|  | ≥ 19 | 0.911 | 1.01 (0.78, 1.31) | 0.859 | 1.02 (0.78, 1.35) | 0.396 | 1.23 (0.76, 1.97) |
| Age at menopause, y | 50 | 0.394 |  | 0.111 |  | 0.624 |  |
|  | ≤ 47 | 0.095 | 0.80 (0.62, 1.04) | 0.636 | 0.93 (0.71, 1.24) | 0.339 | 0.77 (0.46, 1.31) |
|  | 48-49 | 0.937 | 0.99 (0.77, 1.27) | 0.352 | 1.13 (0.87, 1.48) | 0.639 | 1.12 (0.70, 1.81) |
|  | 51-52 | 0.260 | 0.87 (0.67, 1.11) | 0.105 | 0.80 (0.61, 1.05) | 0.982 | 0.99 (0.61, 1.63) |
|  | 53-54 | 0.569 | 0.92 (0.70, 1.21) | 0.987 | 1.00 (0.75, 1.34) | 0.646 | 1.13 (0.67, 1.92) |
|  | ≥ 55 | 0.837 | 1.03 (0.77, 1.38) | 0.414 | 1.13 (0.84, 1.54) | 0.471 | 0.81 (0.45, 1.45) |
| Cycle length, d | 28-30 | 0.051 |  | 0.078 |  | 0.815 |  |
|  | ≤ 27 | 0.017 | 0.64 (0.45, 0.92) | 0.934 | 0.98 (0.66, 1.46) | 0.748 | 0.88 (0.42, 1.87) |
|  | ≥ 31 | 0.528 | 0.91 (0.67, 1.23) | 0.025 | 1.43 (1.05, 1.96) | 0.597 | 1.16 (0.67, 1.99) |
| Bleeding duration, d | 4-6 | 0.680 |  | 0.354 |  | 0.285 |  |
|  | ≤ 3 | 0.469 | 1.08 (0.88, 1.31) | 0.288 | 0.89 (0.72, 1.10) | 0.114 | 1.33 (0.93, 1.89) |
|  | ≥ 7 | 0.756 | 0.97 (0.80, 1.18) | 0.234 | 0.88 (0.71, 1.09) | 0.756 | 1.07 (0.72, 1.58) |
| Way of menopause | | 0.767 | 0.95 (0.70, 1.31) | 0.655 | 1.08 (0.77, 1.51) | 0.084 | 1.67 (0.93, 2.98) |
| Age at enrollment | | < 0.0001 | 1.70 (1.52, 1.91) | 0.053 | 1.13 (1.00, 1.27) | 0.958 | 0.99 (0.80, 1.24) |
| Marital status | | 0.034 | 1.34 (1.02, 1.75) | 0.275 | 1.16 (0.89, 1.52) | 0.199 | 1.34 (0.86, 2.11) |
| Occupational type | | 0.158 | 0.92 (0.82, 1.03) | 0.152 | 1.10 (0.97, 1.24) | 0.481 | 0.93 (0.75, 1.14) |
| Highest educational attainment | | 0.001 | 0.86 (0.79, 0.94) | 0.003 | 0.86 (0.78, 0.95) | 0.128 | 0.85 (0.69, 1.05) |
| Family history of hypertension | | < 0.0001 | 2.29 (1.87, 2.81) | 0.947 | 0.99 (0.80, 1.23) | 0.264 | 1.24 (0.85, 1.82) |
| Family history of diabetes | | 0.019 | 0.70 (0.52, 0.94) | < 0.0001 | 3.37 (2.49, 4.55) | 0.639 | 1.16 (0.62, 2.20) |
| Smoking | | 0.113 | 0.79 (0.59, 1.06) | 0.034 | 0.71 (0.52, 0.97) | 0.407 | 1.22 (0.76, 1.98) |
| Alcohol consumption | | 0.860 | 1.05 (0.61, 1.80) | 0.385 | 1.28 (0.73, 2.26) | 0.110 | 0.20 (0.03, 1.45) |
| Tea consumption | | 0.046 | 0.82 (0.67, 1.00) | 0.371 | 0.91 (0.74, 1.12) | 0.120 | 1.38 (0.92, 2.07) |
| Meat | | 0.013 | 1.34 (1.07, 1.69) | 0.009 | 1.39 (1.09, 1.79) | 0.298 | 0.79 (0.51, 1.23) |
| Viscus | | 0.591 | 1.08 (0.81, 1.44) | 0.260 | 0.84 (0.61, 1.14) | 0.240 | 0.70 (0.39, 1.26) |
| Vegetable | | 0.221 | 1.12 (0.93, 1.34) | 0.455 | 0.93 (0.76, 1.13) | 0.534 | 0.89 (0.63, 1.27) |
| Fish | | 0.001 | 0.66 (0.52, 0.84) | 0.163 | 0.83 (0.64, 1.08) | 0.764 | 1.07 (0.68, 1.70) |
| Salty Food | | 0.060 | 1.18 (0.99, 1.40) | 0.903 | 1.01 (0.84, 1.22) | 0.527 | 1.11 (0.80, 1.53) |
| Plant oil | | 0.657 | 1.04 (0.88, 1.23) | 0.448 | 1.07 (0.90, 1.28) | 0.144 | 0.79 (0.57, 1.08) |
| Animal fat | | 0.245 | 0.89 (0.74, 1.08) | 0.187 | 0.87 (0.71, 1.07) | 0.994 | 1.00 (0.71, 1.41) |

**Supplementary Table S3. Logistic regression analysis of associations between FRFs and hypertension, DM, and LQTc (model 3).**

| Variable | | Hypertension | | DM | | LQTc | |
| --- | --- | --- | --- | --- | --- | --- | --- |
|  |  | *p* | OR (95% CI) | *p* | OR (95% CI) | *p* | OR (95% CI) |
| BMI, kg/m ^2^ | < 24 | < 0.0001 |  | 0.616 |  | 0.068 |  |
|  | 24-27 | < 0.0001 | 1.69 (1.39, 2.04) | 0.363 | 1.10 (0.90, 1.35) | 0.061 | 1.46 (0.98, 2.19) |
|  | ≥ 28 | < 0.0001 | 2.34 (1.75, 3.12) | 0.841 | 1.03 (0.77, 1.39) | 0.025 | 1.84 (1.08, 3.12) |
| WC, cm | < 80 | 0.029 |  | < 0.0001 |  | 0.592 |  |
|  | 80-89 | 0.038 | 1.23 (1.01, 1.49) | < 0.0001 | 1.75 (1.40, 2.19) | 0.967 | 0.99 (0.65, 1.52) |
|  | ≥ 90 | 0.010 | 1.41 (1.08, 1.83) | < 0.0001 | 2.39 (1.80, 3.17) | 0.471 | 1.21 (0.72, 2.04) |
| TC | | 0.020 | 1.28 (1.04, 1.56) | 0.612 | 1.06 (0.85, 1.32) | 0.187 | 0.73 (0.46, 1.16) |
| TG | | < 0.0001 | 1.48 (1.26, 1.75) | < 0.0001 | 1.90 (1.60, 2.25) | 0.312 | 1.18 (0.86, 1.62) |
| UA | | < 0.0001 | 1.39 (1.16, 1.66) | 0.743 | 0.97 (0.80, 1.17) | 0.176 | 1.26 (0.90, 1.76) |
| LDL-C | | 0.702 | 0.96 (0.76, 1.20) | 0.304 | 1.14 (0.89, 1.45) | 0.198 | 1.36 (0.85, 2.18) |
| HDL-C | | 0.375 | 1.16 (0.84, 1.59) | 0.006 | 1.55 (1.13, 2.12) | 0.581 | 0.85 (0.49, 1.49) |

**Supplementary Table S3. Continued**. Abbreviations: y, years; d, days; FRFs, female reproductive factors; DM, type 2 diabetes mellitus; LQTc, long heart-rate-corrected QT interval; BMI, body mass index; WC, Waist circumference; TC, total cholesterol; HDL-C, high-density lipoprotein cholesterol; LDL-C, low-density lipoprotein cholesterol; TG, triglycerides; UA, uric acid.
